# Realization of a Test Tool for Diagnosis of Contact Resistance and Measurement of Selected Types of Conductive Materials

**DOI:** 10.3390/s23135867

**Published:** 2023-06-24

**Authors:** Petr Kacor, Petr Bernat, Tomas Mlcak, Leopold Hrabovsky

**Affiliations:** 1 Department of Electrical Power Engineering at VSB, Technical University of Ostrava, 17. listopadu 2172/15, 70800 Ostrava-Poruba, Czech Republic; petr.bernat@vsb.cz; 2 Department of Electrical Engineering at VSB, Technical University of Ostrava, 17. listopadu 2172/15, 70800 Ostrava-Poruba, Czech Republic; tomas.mlcak@vsb.cz; 3 Department of Machine and Industrial Design at VSB, Technical University of Ostrava, 17. listopadu 2172/15, 70800 Ostrava-Poruba, Czech Republic; leopold.hrabovsky@vsb.cz

**Keywords:** electrical contact, specific resistivity, contact resistance, constriction resistance, fritting, diagnostic, crossed bars, measurement

## Abstract

Contact connections in electrical machines and apparatus are important elements in the whole power supply network and a high level of reliability is expected there. Contact resistance is a fundamental criterion in the design of an electrical contact or contact system. The contact resistance should be as low as possible to minimize losses due to the current passage and the related heating of the contact connection. The value of the contact resistance depends on the material used, the value of the applied force, the type of contact, and, last but not least, the quality of the surface and chemical layers. In this paper, an initial diagnosis of the contact material is performed based on the determination of the sample’s specific resistivity by the four-wire method and the evaluation of the measurement uncertainty. The work is followed by the design of a testing device that uses crossed bars to measure the change in contact resistance as a function of the magnitude of the applied force. An analysis of the sample mounting method is performed here using FEM simulations of the current field and shows the interaction between the holder and the sample in terms of current line transfer. The proposed system is then used for experimental measurements of the material-dependent coefficient *K_C_* for verification of existing or newly developed materials in electrical engineering, where the values of the *K_C_* coefficient are not known. Finally, the paper also deals with the measurement of fritting voltage for individual contact pairs having surface quality corresponding to brushing.

## 1. Introduction

Electrical apparatus plays a fundamental role in maintaining a reliable distribution of electrical energy and are an essential part of any transmission and distribution power network. The degree of reliability is greatly dependent on the reliability of the switching elements, which, among other things, provide the control and operation of the electrical power network and generally ensure the transport and distribution of the electric energy generated in the power plants to the consumers safely and reliably. Thus, the reliability of the electrical energy delivery and the reliability of the electrical apparatus are interrelated domains [1,2].

From the point of view of operational safety, the electrical apparatus must in general ensure galvanic insulation between the consumption and the supply. Fault situations such as overloads, overvoltage, or short circuits sometimes occur in networks and the electrical apparatus must also fulfill a safety and protection function [3,4].

In industrial applications, we also often find apparatus systems for switching or reconnecting transformer taps and voltage controls. In recent decades the trend is to replace these systems with power electronic equipment, although we still find applications with classical mechanical switching [5,6].

A shared feature of all the above switching structures is the contact system one of the most important parts of any switching apparatus [7]. In the contact system, there is a mutual movement of the current paths, and the switching on and off of the energy flow takes place. The interruption of the conduction path can be realized under different operating conditions, e.g., with current, without current, at different voltages, with high switching density, etc. [7,8]. In general, the requirements for contacts can be characterized as follows [9]:

- Low contacts resistance;
- High durability to mechanical wear (abrasion);
- High resistance to welding;
- High resistance to electric arc burning;
- Optimal influence on the development of deionization processes after extinguishing the switching arc (good switching capability).

A low contact resistance is an important factor in the choice of contact material. From the current conduction point of view, the contact material should have the highest possible conductivity. Such materials exist (Ag, Cu), but they are usually mechanically soft or sensitive to surface layers or the formation of welds [9,10]. Tungsten or molybdenum, for example, has a high withstand capability against arcing. However, low electrical conductivity is typical for these materials and they are not suitable for direct contact applications [10,11].

**Figure 1:**
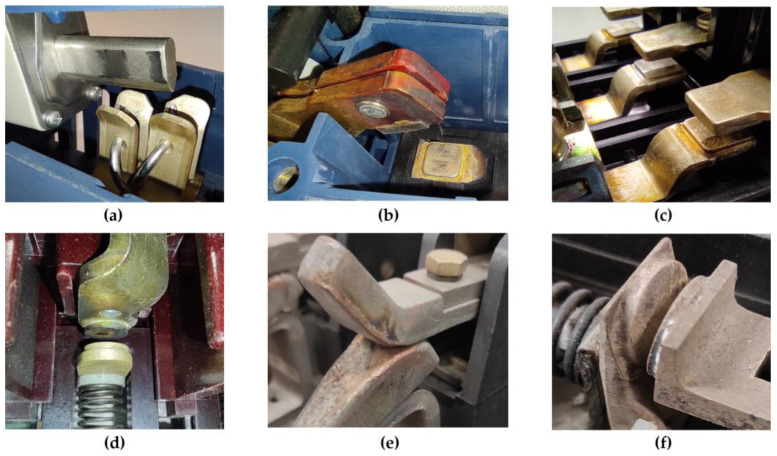
Examples of power apparatus contact systems: (**a**) Knife contact of fuse and fuse disconnector; (**b**) Low-voltage circuit breaker; (**c**) AC power motor contactor; (**d**) Old-style AC power motor contactor; (**e**) DC contactor without extinguishing chamber; (**f**) AC power switch.

Fulfillment of all the above conditions and defined requirements for contacts are often contradictory. The problem of contact design is approached according to the application of the electrical apparatus [9,12]. Examples of typical contact shapes for electrical equipment are shown in Figure 1.

With the development of electrical engineering, higher and higher requirements are placed on contact systems. In particular, reliability and a long lifetime are required. The description of electrical contacts implemented e.g., in busbar connections, switchgear systems, or electrical apparatus, and the search for interdependencies between materials, surface layers, surface quality, and wear, have been studied by many authors worldwide for several decades [10,12,13,14,15,16,17,18,19]. Currently, contact design and development use complex numerical models and simulations that analyze not only the mechanical (roughness, elasticity, plasticity) and electrical (conductivity, contact bridges, chemical layers) aspects of contacts but also deal with thermal aspects (heating, cooling, softening, and welding voltage). Even as the parameters needed to design a reliable contact system are continuously improved, we still cannot avoid validation measurements that verify the results of these simulations [20,21,22,23,24].

Measurement of contact resistance is necessary for the testing and development of new contact materials required for various working conditions and applications. The value of contact resistance is a crucial criterion in the design of a contact system. It should be minimal to reduce heat loss. The magnitude of the contact resistance is affected by a series of factors where the most important ones are the material properties (electrical conductivity), the existence of impurities, oxides, and chemical layers, the shape and type of contact (point, line, and surface) and the applied contact force (contact pressure) [9,10,24].

Online contact diagnostics can be performed after the unit is manufactured or while the unit is already operating in the substation. The overall condition of the switching apparatus, including its current paths and connecting terminals, is evaluated. The static method consists in injecting direct current in the switched-on state of the device and measuring the voltage drop, looking for changes in parameters during switching operations. [25,26,27]. The dynamic method injects a high value of direct current (more than 100 A) to ignite an electric arc. Measuring the waveform of the current and voltage during the switching of the device is the base of this method. The contact surface erosion as well as the shortening of arc contacts can be detected in time [28,29].

In general, offline diagnostics can be considered a situation where the measurement of diagnostic parameters is carried out on individual components of the device in a disassembled state. Using offline diagnostics, the quality of the contact connection can be determined, for example, by measuring the contact resistance on recently manufactured or already used contacts. The influence of switching frequency on the wear of the contact surfaces and the range of change of the contact resistance *Rs* can be determined in this way [12,20,30,31].

One of the possibilities to perform offline diagnosis is the evaluation of contact resistance, e.g., using crossed bars [9,10,12]. The advantage lies in capturing the ratio between the contact resistance *R_S_* and the applied force *F*. With the knowledge of other parameters such as the surface hardness and the modulus of elasticity of the contact material, it is possible to determine, for example, the average resistivity of the surface layers. The importance of this type of measurement is evident in the development of new or optimized contact materials or metal alloys [32,33,34,35].

Contact properties are a rather complicated function and complex dependence of both physical properties (e.g., density, chemist, hardness, electrical/thermal conductivity, structure, etc.) and operating conditions (voltage, current, frequency, power factor, frequency, contact on/off velocity and force, etc.) [11,12]. Specialized techniques and equipment are required to evaluate these contact properties. In particular, contact resistance, opening, and closing velocity effect, contact bounce, contact welding measurement, erosion, etc. are evaluated. In contact resistance measurements, two approaches are used, namely, crossed-bar arrangement or gold probe measurements under very low mechanical and electrical load to avoid any mechanical or electrical damage to the surface layer [36,37,38].

The main motivation for the research activity was the need to create a measurement system that can verify the electrical parameters of newly developed material structures and alloys. These new structures were formed by combining highly electric conductive materials Al/Cu by rotary swaging, rolling, or extrusion. The cross-section of these materials can be of sandwich composition or the secondary material is embedded in the shape of segments, bars, or tubes. The manufacturing process conditions and the method of additional heat treatment are also different [39,40].

Such materials have good potential in electrical applications in the design of electrical machines (hybrid rotors of asynchronous motors) and apparatus (bus bars, input terminals, parts of conducting ways or contacts) [41,42,43,44]. The partial substitution of Cu by Al generally leads to a reduction in weight while improving the mechanical and performance properties of the solid conductor without significantly reducing its electrical conductivity.

Authors often cover the mechanical or thermal properties of newly developed structures in their studies, but the electrical parameters of these materials are rarely or only occasionally discussed [45,46]. This offers the opportunity to fill a gap in knowledge of the specific electrical parameters of Al/Cu structures and to use electrical resistivity measurements to determine important design parameters required in electrical engineering practice. As a secondary step, the properties of these materials concerning contact coupling can then be also validated.

In the reference literature, in most cases, only conceptual diagrams of the testing device or details focusing on the contact pair appear, but the overall view of the measuring system with its detailed description is missing and reproducibility is thus impossible [20,22,24,25]. Therefore, this paper aims to analyze and implement the design of a verification tool and in the first phase of the work to perform electrical conductivity measurements with an extension to the measurement of contact resistance parameters on selected materials.

Concerning the current state of the art, the paper presents a possible way to identify existing materials or newly developed alloys or structures. The obtained measurement outputs can be directly used in the design or optimization of contact systems of switching apparatus. The main objective and new contribution of this paper are then to investigate the properties of materials in the initial stage of production and the influence of subsequent heat treatment. The construction of the test system was designed, verified, and modified, and based on the comparison of experimental results of contact resistance with available theoretical results, limits and flaws of the device were found.

## 2. Materials and Methods

The analytical models for calculating the contact resistance are based on the fact that the current between two electrically conductive components passes through a defined metallic reduced contact surface (a-spot) with a specific geometry. The current that passes through this conductive contact spot causes a so-called constriction resistance *Rc* due to the increased current density. There are different models for the description of the constriction resistance of electrical contact, but the generally accepted model for the calculation of electrical contacts is the ellipsoidal model according to Holm [9,10,11]:

(1) $$R_{C}=\frac{\rho }{2\pi \cdot a}tan^{-1}\left(\frac{\sqrt{\mu }}{a}\right)$$

where *ρ* (Ω·m) is the resistivity of the contact material and *a* (m) is the radius of the contact surface. The parameter *µ* defines the distance of the vertical semi-axis of the ellipsoidal potential surface from the contact surface. Considering the dimensions of the contact body in comparison to the dimensions of the contact surface, it can then be assumed that $\sqrt{\mu }$ → ∞. For the total constriction resistance of the two contacts, it can be written:

(2) $$R_{C}=\frac{\rho }{2a}$$

Because metals are not clean, the passage of electric current can be affected by thin layers of oxides, sulfides, and other inorganic substances that are usually present on the contact surface of metals. As a result, the total contacts resistance *R_S_* of the connection is the sum of the constricted resistance *R_C_* and the film resistance *R_f_* [10,12]:

(3) $$R_{S}=R_{C}+R_{f}$$

The film resistance *R_f_* can be calculated:

(4) $$R_{f}=\frac{\sigma_{f}}{\pi \cdot a^{2}}$$

where *σ_f_* (Ω·m^2^) is the resistance per film area.

To determine the size of the contact area *a* (a-spot) at the contact of two elements, the deformation, and tension due to the applied force can be calculated for the purely elastic behavior of the material according to Hertz’s theory. The assumption is the formation of a circular contact area [10,11]. The radius of the contact area for the shape of the sphere-plate contacts or the case of crossed cylindrical bars can be calculated as:

(5) $$a=1.11\sqrt[{3}]{\frac{F\cdot r}{E}}$$

where *F* (N) is the contact force, *r* (m) is the radius of the cross bars and *E* (Pa) is the tensile modulus of elasticity. In addition to the elasticity condition, the Formula (5) is derived with the assumption that there is no friction and the bodies have smooth and spherical surfaces.

If the contact force *F* is larger, a combination of elastic and plastic deformation or purely plastic deformation may occur at the location of contact. For practical purposes, it is appropriate to consider only either pure elastic or pure plastic deformation. The combination of both deformations in the design of contacts then leads to difficult calculations and considerations of an extensive character [10,11]. For the case of plastic deformation, the formula for the contact area radius a-spot of the form:

(6) $$a=\sqrt{\frac{F}{\pi \cdot \sigma_{Pd}}}$$

where *σ_Pd_* (Pa) is the compressive strength (contact hardness). By substituting the above Equations (5) and (6) into Equation (2) we obtain the final relations for the calculation of the constricted resistance for elastic contact (indexed as CE) and contact with plastic deformation (indexed as CP):

(7) $$R_{CE}=\frac{\rho }{2.22\cdot \sqrt[{3}]{\frac{r}{E}}}\cdot F^{-\frac{1}{3}}$$

(8) $$R_{CP}=\frac{\rho }{2}\cdot \sqrt{\pi \cdot \sigma_{Pd}}\cdot F^{-\frac{1}{2}}$$

Thus, in the case of elastic deformation, the contact resistance is a function of the force with an exponent of ⅓ and in the case of plastic deformation with an exponent of ½. The choice of the correct relationship for calculating the contact resistance is difficult and for practical purposes should be chosen with care.

In the practical calculation of the contact resistance *R_CO_*, the empirically derived relationship between contact resistance and contact force is preferably used [7,8]:

(9) $$R_{CO}=K_{C}\cdot F^{-n}$$

The coefficient *K_C_* generally depends on the contact material, the type of machining, and the condition of the contact surface and also includes the influence of surface layers. The exponent *n* is then chosen according to the type of contact (shape-dependent exponent of the contact force). Empirical determination of the contact resistance is also useful for flat or line contacts that cannot be easily calculated analytically [47,48,49].

Typical values of the material-dependent coefficient *K_C_* and the contact force exponent *n* are given in Table 1; the label LC denotes Low Current contacts type [8,50].

Since the coefficient *K_C_* includes the effect of surface layers and takes into account the character of the contact force, Equation (9) gives higher values of resistance with load in comparison with theoretical relationships. The advantage of the empirically derived coefficient *K_C_* is that its value can be determined also for the combination of two different contact materials [8,9]. Table 1 focuses on power contact systems of electrical apparatus, where higher contact forces and plastic deformation are considered. For all types of contact interfaces, the force exponent *n* is always at least equal to ½ or higher.

### 2.1. Design and Construction of the Measuring System

**Figure 2:**
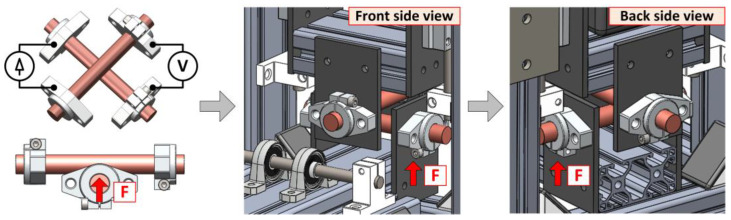
Basic design layout of testing tool with initial CAD design (The red arrow shows the direction of the applied force F).

The basic concept of the contact resistance measurement system is based on the recommendations of Mr. Holm and uses a cross-bar arrangement of the sample. This arrangement is advantageous due to the expected shapes of the material samples to test, but mainly because it eliminates the influence of the solid body resistance of the bar [10]. Figure 2 shows the basic layout and conceptual CAD design.

The designed testing tool assumes the mounting of samples by holders which also operate as an electric current input and at the opposite side for voltage sensing. The insulating side plates to mount the contact holders are made of FR4 material. The measuring system is assembled from industrial aluminum profiles, allowing a large variation in shape. All construction elements are sufficiently rigid and the connections are made with bolts and tightening nuts.

**Figure 3:**
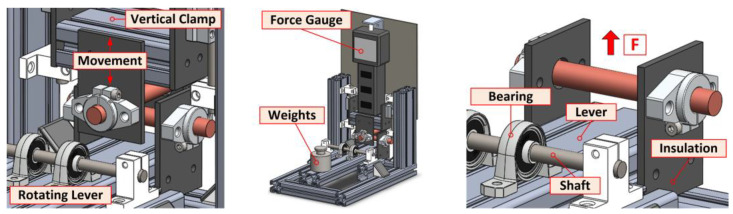
Detailed view of crossed bars and location of force gauge on testing tool (CAD design).

The upper bar of the tested material is mounted in a holder on a vertically sliding base, on top of which a force gauge is inserted. The self-weight of the base and the force gauge is balanced by springs on both sides. The lower bar holder is part of a lever freely rotated in bearings around the shaft. A weight placed on the opposite side of the lever then generates the contact force, see Figure 3.

**Figure 4:**
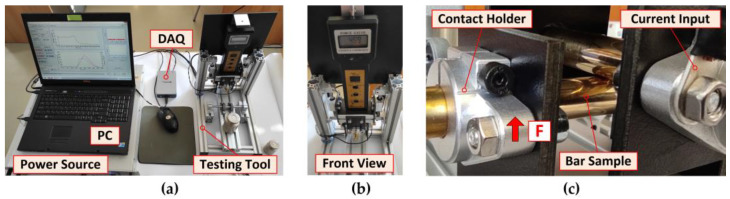
Assembled measuring system and detail of crossed bars in holders: (**a**) Overall view; (**b**) Front view; (**c**) Detail of crossed bars and sample holders.

The inputs of the current clamps and the outputs of the voltage sensors are realized directly on the holders of the bars. The samples are mounted relatively freely in the holders so that they can be rotated during the contact resistance measurement and the “fresh contact surfaces” can be easily adjusted against each other. The complete realization of the measuring system with the stored weights and the detail of the brass crossing bars is shown in Figure 4a–c.

The design of the measuring system assumed that there is no perfect contact between the holder and the measured sample over the entire circumferential surface. The supply current into the bar enters only from one side of the holder. This causes a deformation of the current lines in the bar sample, which is transferred from the holder space to the expected contact point (mid-length of the bar). The deformation of the current density decreases with distance from the holder, but we do not know at this time how strong and what effect the geometrical dimensions of the sample and its electrical conductivity have on this.

**Figure 5:**
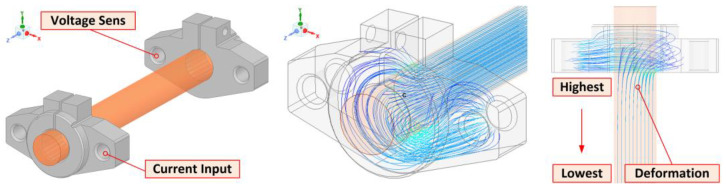
FEM simulation of current line deformation due to weak holder surface connection.

The requirement is that the deformation of the current lines due to the holder is not transferred to the point of contact. In other words, we were looking for the minimum necessary sample length *L_Smin_*. FEM simulation of the current field distribution was used to solve this problem, see Figure 5.

Figure 5 presents the FEM model of the holders and the attached bar. The plotted current lines show the extreme deformation at the point where the attached bar leaves the holder. This deformation of the current lines extends to the point of assumed contact with the second bar.

**Figure 6:**
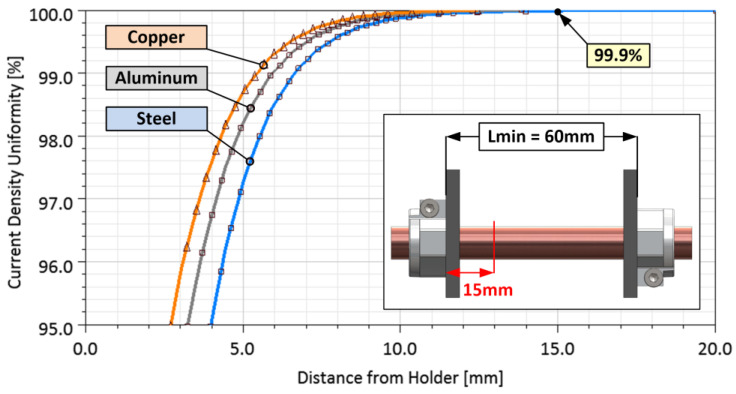
Degree of deformation of current lines with increasing distance from the holder.

The degree of deformation of current lines is shown in the graph in Figure 6. Here the dependence of the current density *J* in the axial direction of the bar is plotted. The values are normalized with respect to the uniformity of the current density distribution at a very far point from the holder and expressed as a percentage. Three materials, copper, aluminum, and steel, were simulated. In the case of copper, the deformation of the current lines decreases at a much faster rate than that of the steel sample.

At a distance of *L_X_* = 15 mm from the holder, the uniformity of the current density reaches about 99.9%. It can be considered homogeneously distributed over the entire cross-section. This length is then in fact equal to half of the minimum necessary distance between the holders. For safety, the real holder distance is doubled (*L_min_* = 60 mm), as shown in Figure 6.

**Figure 7:**
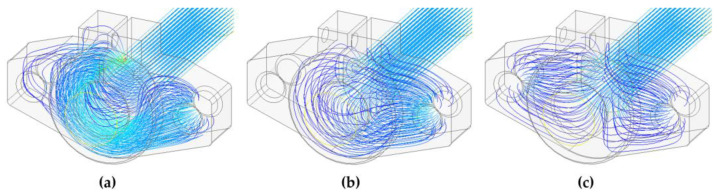
Selected simulations of current path entering the sample: (**a**) At the longitudinal edge of the holder only; (**b**) Across the entire contact area of the sample and the holder; (**c**) Across the entire contact area of the sample and the two-sided power supply of the holder.

The issue of current lines through different materials is discussed quite well in [11]. The deformation of the current lines is caused by the rapid change in geometry as well as the value of the electrical conductivity of the material, including the character of the contact between the holder and the sample. To complete the design of the sample holding system, Figure 7 shows the current line distribution solution in several considered situations.

Figure 7a shows a state where contact with the sample is ensured only at the longitudinal edge of the holder and simulates an insulating layer on the surface of the sample in contact with the holder. The other cases in Figure 7b,c then show ideal contact over the entire surface and current entering the holder from two sides. In the models, the interface layer between the holder and the sample was not considered, but the insulating region was simulated by inserting a non-conductive area without an air gap.

### 2.2. Samples Identification—Measurement of Electrical Resistivity

In the first step, the identification of the material samples was carried out. An important parameter from this point of view is the electrical resistivity *ρ*, which varies considerably for the considered samples of contact materials. Tabular data can be used for contact design [51], but a more accurate procedure is to measure the resistivity directly on the samples under study.

**Figure 8:**
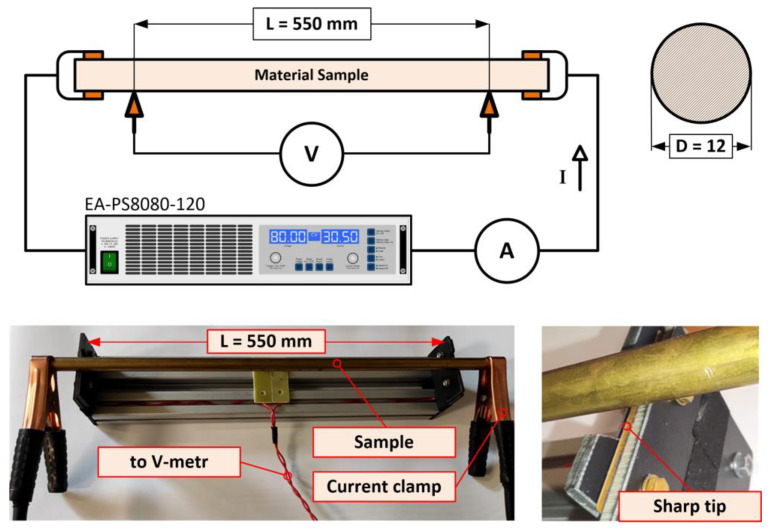
Schematic diagram of the circuit for measuring the resistivity and practical realization.

Ohm’s law and the four-wire method [52] were used to determine the specific resistivity of the material samples. Each sample was supplied with DC using a current-limited EA-PS8080-120 power supply, and the voltage drop *V* between the electrodes connected to the sample surface was measured, see Figure 8. It was considered that the magnitude of the load current would generate only a negligible temperature rise. Due to the diameter of the sample *D* = 12 mm, the current value was set to *I_max_* < 20 A.

The magnitude of the load current *I* when measuring resistance *R* is always a compromise between the voltage drop *V* on the voltage electrodes and the overall temperature rise of the sample Δ*υ*. The value of current was chosen considering adiabatic heating without dissipation of heat to the surroundings. The total sample temperature rise over time can be determined from the:

(10) $$Q=m\cdot c_{m}\cdot \Delta \vartheta =R\cdot I^{2}\cdot t$$

where *Q* (J) is the heat, *m* (kg) is the sample weight, *c_m_* (J·kg^−1^·K^−1^) is the thermal capacity, Δ*υ* (K) is the temperature rise, *R* (Ω) is the sample resistance, *I* (A) is the magnitude of loading current, and *t* (s) is the time of its passing. After the modifications of (9), the temperature rise of the sample can be calculated:

(11) $$\Delta \vartheta =\frac{R\cdot I^{2}}{m\cdot c_{m}}\cdot t=\frac{\rho \cdot I^{2}}{A^{2}\cdot c_{V}}\cdot t$$

where *ρ* (Ω·m) is the specific electrical resistivity, *A* (m^2^) is the sample cross-section and *c_V_* (J·m^−3^·K^−1^) is volumetric thermal capacity. At a constant value of load current, *I* = 20 A, the sample with the lowest electrical conductivity will achieve the highest temperature rise. Therefore, we add the values of conductivity corresponding to 15% IACS (similar to the steel S235JR sample):

(12) $$\Delta \vartheta =\frac{\rho \cdot I^{2}}{A^{2}\cdot c_{V}}\cdot t=\frac{115\cdot 10^{-9}\cdot 20^{2}}{{\left(\frac{\pi \cdot 0.012^{2}}{4}\right)}^{2}\cdot 3.69\cdot 10^{6}}\cdot 1=0.001\;K$$

The result of (12) shows that for the sample with the lowest conductivity (S235JR) when a current of *I* = 20 A is applied, the temperature increases by 0.001 K every second. The time for one measurement and reading of the electrical quantities after the current was switched on never exceeded *t_M_* = 10 s. This was followed by a break of approximately *t_B_* = 120 s to cool down.

Equation (13) was used to calculate the specific electrical resistance and Equation (14) was derived by adding the geometric dimensions of the measured sample:

(13) $$R=\rho \cdot \frac{L}{A}$$

(14) $$\rho =R\cdot \frac{A}{L}=\frac{V\cdot A}{I\cdot L}=\frac{\pi }{4}\cdot \frac{V\cdot D^{2}}{I\cdot L}$$

where *ρ* (Ω·m) is the specific electrical resistivity, *A* (m^2^) is the cross-section of the sample, *V* (V) is the voltage drop, *D* (m) is the diameter of the sample, *I* (A) is the DC supply current and *L* (m) is the spacing between voltage electrodes located on the sample surface.

As this is generally a task of determining the material resistivity *ρ* using indirect measurements, it was necessary to quantify the measurement uncertainties Type A and B for all measured data and then integrate these influences into the final result [53,54]. An example of the evaluation is performed on a brass bar with the label CuZn39Pb3.

#### 2.2.1. Direct Measurement of the Diameter and the Spacing of the Sensing Electrodes

The geometric dimensions of the sample were measured using two Mitutoyo digital calipers. One was used to measure the sample diameter *D* and the other to measure the electrode spacing *L*. A total of ten measurements (*n* = 10) were completed under the same conditions in both cases. The bar diameter *D* was measured at randomly selected points, namely at both ends and in the middle of the sample length.

The measured data were tested for normality using the Shapiro-Wilk test and at a significant level of *α* = 0.05, the hypothesis that the measured statistical sample is normal was confirmed [55]. The estimated value of the final brass bar diameter *D* is given by the arithmetic mean of the individual measurements *D_k_*:

(15) $$\bar{D}=\frac{1}{n}\;\overset{n}{\underset{k=1}{\sum }}D_{,k}$$

The corresponding uncertainty of Type-A associated with the estimation of *D* is determined as the experimental standard deviation of the average. For the case of repeated measurements of *n* ≥ 10 then:

(16) $$u_{A}\left(\bar{D}\right)=\sqrt{\frac{1}{n\left(n-1\right)}\;\sum_{k=1}^{n}{\left(D_{,k}-\bar{D}\right)}^{2}}$$

Equations (15) and (16) are also used for the case of measuring the spacing *L* of the voltage electrodes. The processed data are shown in Table 2.

A certificate is available from the caliper manufacturer that indicates that the instrument has a resolution error of *δ*_1_ = (0.02 + 0.00005·*L*) within the length measurement interval *L* = (0 ÷ 150) mm. This gives a total resolution error of *δ*_1_ = 0.0206 mm when taking into account the diameter of *D* = 12 mm. In Type B uncertainty we also consider operator influences representing the imperfection of the alignment of the measuring instrument for the diameter of the bar, varying pressure of the measuring plates, etc. We consider the magnitude of this error to be approximately equal to the resolution error of the instrument, i.e., *δ*_2_ = 0.021 mm. For both errors *δ*_1_ and *δ*_2,_ we also assume a uniform rectangular distribution under, then [53]:

(17) $$u_{B1}\left(\bar{D}\right)=\frac{\delta_{1}}{\sqrt{3}}\;\;\;\;\;\;\;\;\;\;\;\;\;\;\;\;\;\;\;\;u_{B2}\left(\bar{D}\right)=\frac{\delta_{2}}{\sqrt{3}}$$

The final B-Type standard uncertainty of the bar diameter estimation *D* is calculated as a summarization of both uncertainties:

(18) $$u_{B}\left(\bar{D}\right)=\sqrt{u_{B1}{}^{2}\left(\bar{D}\right)+u_{B2}{}^{2}\left(\bar{D}\right)}$$

The combined uncertainty is finally obtained by adding *u_A_*(*D*) and *u_B_*(*D*) and corresponds to the relation:

(19) $$u_{C}\left(\bar{D}\right)=\sqrt{u_{A}{}^{2}\left(\bar{D}\right)+u_{B}{}^{2}\left(\bar{D}\right)}$$

The relations (16) to (19) are also used to process the values measurements of the electrode spacing *L*. The processed data for diameter *D* and electrode spacing *L* are shown in Table 3.

#### 2.2.2. Direct Measurement of Current I and Voltage Drop V

The procedure for the evaluation of the measured current and voltage is the same as for the determination of the Type A and B uncertainties in Section 2.2.1. For both electrical quantities, the Shapiro-Wilk test was performed, the arithmetic average of 10 sample measurements under the same conditions was determined (15) and the standard deviation was calculated according to (16). The processed data are presented in Table 4.

The current *I* passed through the measured sample was measured with a digital ammeter. TRMS multi-meter, Model ANENG 870 (as the ammeter) with 20,000 counts LCD. The accuracy of this multi-meter for the DC range was ±(0.5% reading + 3 digits) with a resolution of 0.001 A. The voltage *V* at the sensing electrodes was measured with a digital voltmeter. TRMS multi-meter, Model ANENG 870 (as the voltmeter) with 20,000 counts LCD and input impedance > 10 MOhm.

The accuracy of this multi-meter for the DC millivoltage range was ±(0.05% reading + 3 digits) with a resolution of 0.001 mV. The following relationship was used to calculate the measurement uncertainty of the digital meter:

(20) $$u_{B}\left(\bar{V},\bar{I}\right)=\pm \left(\frac{MV}{100}\cdot \left|\delta_{RDG}\right|+DIGS\cdot RES\right)$$

where *MV* is the measured value, *DIGS* is the number of digits and *RES* is the resolution of multi-meter at selected range. The processed data are presented in Table 5.

The specific electrical resistivity *ρ* of the measured sample is calculated using the derived relation (14) by adding the values from Table 3 and Table 5:

(21) $$\rho =\frac{\pi }{4}\cdot \frac{V\cdot D^{2}}{I\cdot L}=\frac{\pi }{4}\cdot \frac{6.39\cdot 10^{-3}\cdot {\left(11.98\cdot 10^{-3}\right)}^{2}}{19.14\cdot 550.14\cdot 10^{-3}}=68.41\cdot 10^{-9}\;\Omega \cdot m$$

As the resistivity of the sample is determined by indirect measurement, it is necessary to combine all the estimated uncertainties in the current, voltage, and geometric dimensions of a sample by applying the law of uncertainty propagation to the measurement model used [53,54]. We did not consider any covariance and the measured values of the voltmeter, ammeter, and calipers were not correlated.

The task was to determine the individual sensitivity coefficients Ai using partial derivatives of all variables:

(22) $$\frac{\partial \rho }{\partial D}=\frac{\pi }{2}\cdot \frac{V\cdot D}{I\cdot L}\;\;\;\;\;\;\;\;\;\;\;\;\;\frac{\partial \rho }{\partial L}=-\frac{\pi }{4}\cdot \frac{V\cdot D^{2}}{I\cdot L^{2}}\;\;\;\;\;\;\;\;\;\;\;\;\frac{\partial \rho }{\partial I}=-\frac{\pi }{4}\cdot \frac{V\cdot D^{2}}{I^{2}\cdot L}\;\;\;\;\;\;\;\;\;\;\;\;\frac{\partial \rho }{\partial V}=\frac{\pi }{4}\cdot \frac{D^{2}}{I\cdot L}$$

The final standard uncertainty of the specific resistance of the bar sample is given by combining all these uncertainties as follows:

(23) $$u_{\rho }=\sqrt{{\left(\frac{\partial \rho }{\partial D}\right)}^{2}\cdot u_{c}^{2}\left(D\right)+{\left(\frac{\partial \rho }{\partial L}\right)}^{2}\cdot u_{c}^{2}\left(L\right)+{\left(\frac{\partial \rho }{\partial I}\right)}^{2}\cdot u_{c}^{2}\left(I\right)+{\left(\frac{\partial \rho }{\partial V}\right)}^{2}\cdot u_{c}^{2}\left(V\right)}$$

The processed data are shown in Table 6.

The remaining material samples, ETC copper, aluminum, and steel bar, were measured in a similar procedure. The summary results of the specific electrical resistivity measurements are shown in Table 7. This table also includes the modulus of elasticity values collected from the material supplier’s data sheets. For comparison, tabulated values of specific resistivity obtained from reference literature are added here [11,51].

### 2.3. Preparing Samples for Contact Resistance Measurements

**Figure 9:**
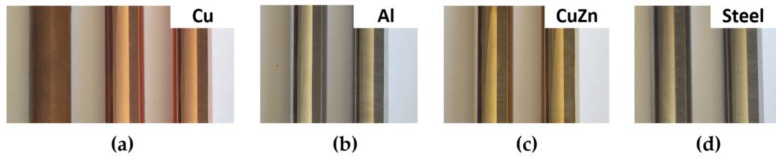
Example of the surfaces of samples: (**a**) Copper; (**b**) Aluminum; (**c**) Brass; (**d**) Steel.

All samples were cut to the required length once the resistivity measurements had been completed. Samples with heavy surface contamination were degreased. The surface was sanded first with coarse and then fine sandpaper. The final mechanical surface treatment was brushing. Immediately after the surface treatment, a layer of technical lubricant was applied to the samples to protect their surface from external influences, see Figure 9.

The difference in surface quality between the original copper bar sample and the sample after brushing is shown in Figure 9a. The surfaces of the aluminum, brass, and steel bar samples were prepared in the same way, see Figure 8b–d. The sample surfaces have much less roughness and contamination compared to the contact surfaces commonly found in electrical power apparatus, see Figure 1 and Figure 9.

### 2.4. Procedure for Measuring the Contact Resistance

The measured sample material was mounted in the holders. The contact force *F* was set using a weight inserted on the arm of the device and measured with a force gauge. The verification of the contact force was carried out without an electric current. A power supply was then connected with the current limited to *I* = 1 A and the magnitude of the voltage drop V across the contacts was measured simultaneously. After measuring the voltage drop, the power supply was briefly switched off and then on again. The measurements were carried out 10 times in this way. After this cycle, the contacts were unloaded and the sample material in both holders was rotated. After the contact force was applied again, the measurement cycle was repeated.

A total of 5 contact positions for the same magnitude of contact force *F* were measured using this sequence. Supply current *I* and voltage *V* were measured using a DAQ card and LabView application with continuous recording of measured values. After the measuring cycle, the contact force *F* was changed and the procedure was repeated in the total range of loading *F* = {0.1, 0.5, 1, 2, 5, 10} N.

**Figure 10:**
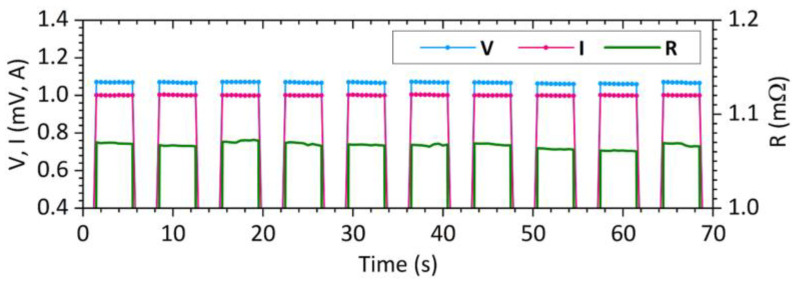
Time dependence of current, voltage and resistance during measurement, *F* = 5 N.

The waveform of the captured voltage drop *V* and current *I* during the measurement for a contact force *F* = 5 N on the brass bars is shown in Figure 10. All the material samples used were measured using the same procedure.

## 3. Results

The measured data were processed for each material and the results of the contact resistance plotted on a graph *R_CO_* = *K_C_ F^−n^* in logarithmic scale. The values of the coefficient *Kc* and exponent *n* for all contact material combinations are shown in Table 8.

**Figure 11:**
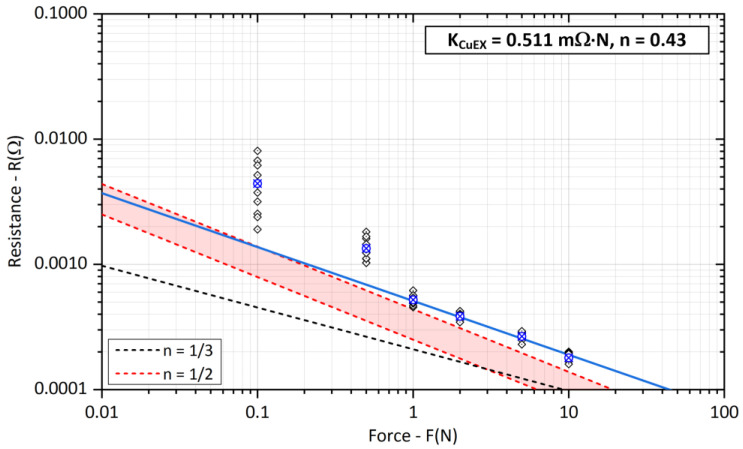
Experimental evaluation of the *K_Cu_* coefficient and exponent *n* for copper bars (The blue cross represents the average value of the measured points).

The dependence of the contact resistance *R_SCu_* on the applied contact force *F* for crossed copper bars is shown in Figure 11.

The black dashed line shows the dependence under pure elastic deformation of the contact point using (7) with values of the resistivity and modulus of elasticity parameters from Table 7. The red dashed lines indicate the range of contact resistance extracted from Table 1, where a full plastic deformation of the contact area is assumed. The exponent *n* here equals −½ and dependencies were derived from measurements on real contacts of power electrical connections.

Finally, the blue line corresponds to the experimentally determined parameters of the coefficient *K_CuEX_* with associated exponent *n*. For lower contact forces *F* = 0.1 N and 0.5 N, there was a significant deviation in the measured values and these results were not included in the overall result. Measurements at low contact forces *F* < 1 N were also very sensitive to small vibrations and shaking, we decided to exclude them from all evaluations. Thus, the blue line was obtained by interpolating the average values of the experimentally measured resistances in the range of *F* = {1, 2, 5, 10} N.

**Figure 12:**
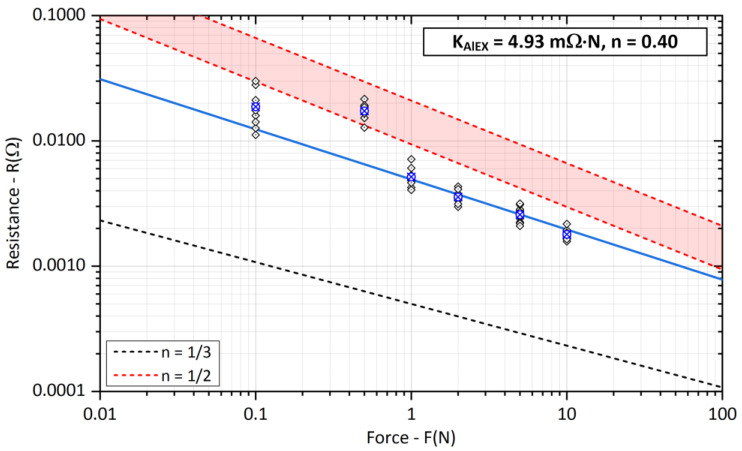
Experimental evaluation of the *K_Al_* coefficient and exponent *n* for aluminum bars (The blue cross represents the average value of the measured points).

Contact resistance *R_SAl_* measurements on aluminum crossed bars were relatively difficult to perform, the results of which are shown in Figure 12. The experimentally determined coefficient *K_AlEX_* reaches lower values when compared to the data in Table 1, the exponent *n* adjusts the slope of the line more to the elastic form of deformation. The blue line was obtained by interpolating the average values of the experimentally measured resistances in the range of *F* = {1, 2, 5, 10} N.

For small contact forces *F* < 1 N, there was also a significant variance in the measured resistance values. In some measurements, the contact resistance even showed a value of *R_C_* → ∞, although the force gauge indicated a load of *F* = 0.5 N. This is probably due to the very hard oxides Al_2_O_3_ on the surface of the aluminum bars. Treatment with technical grease immediately after finishing the contact surface did not help either. When the measured bars were touched, there was no friction of the contact surfaces, but only direct contact, thus the removal of the surface layers was ineffective and fully affected the contact resistance value.

**Figure 13:**
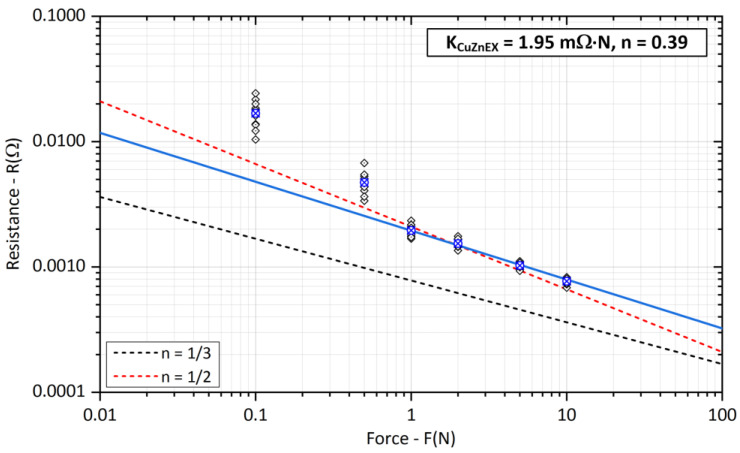
Experimental evaluation of the *K_CuZn_* coefficient and exponent *n* for brass bars (The blue cross represents the average value of the measured points).

The results of the contact resistance *R_SCuZn_* measurements on brass-crossed bars are shown in Figure 13. Compared to the measurements on aluminum bars, the experimentally determined coefficient *K_CuZnEX_* is smaller, although the electrical conductivity of brass is much lower. This is confirmed by the values obtained from the literature reported in [8] and captured in Table 1. Here, the *K_CuZnEX_* coefficient reaches approximately 2.5 times lower values than *K_AlEX_* for aluminum, and the surface layers break down significantly better.

**Figure 14:**
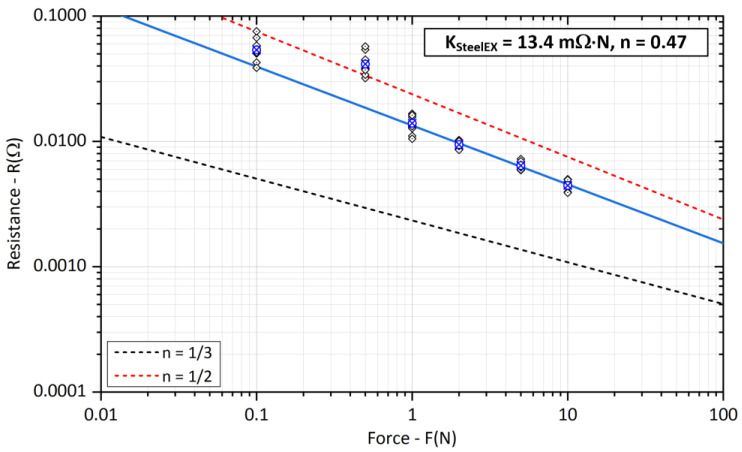
Experimental evaluation of the *K_Steel_* coefficient and exponent *n* for steel bars (The blue cross represents the average value of the measured points).

The measurements of contact resistance *R_SSteel_* on steel crossed bars are shown in Figure 14. Compared to the other types of measured samples, this is a very hard material with the lowest electrical conductivity (12% IACS). The value of coefficient *K_SteelEX_* shows better results than Table 1. The value of the exponent *n* is the closest to the plastic deformation of the contact area of all tested contact materials.

### Measurement of Fritting Voltage and Change of Contact Resistance with Current Loading

In the next step of the measurement, the contact materials were loaded with a higher current value, which reached the magnitude of *I_max_* = 120 A. The waveform and the overall evolution of the voltage drop and the change of the contact resistance *R_S_* after load removal were monitored.

**Figure 15:**
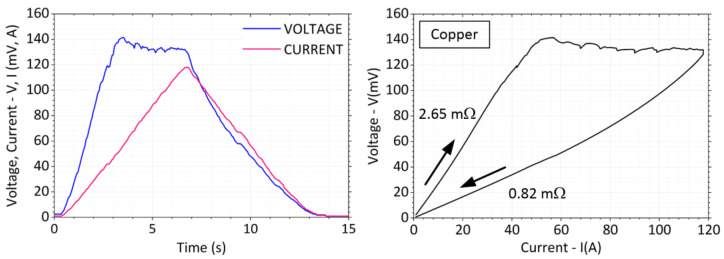
Current and voltage waveform with *V-I* characteristic for copper bars.

The total measurement time was in the range of *t* = 15 s. There was an increasing current in the first half and a decrease in the second half of the interval. Figure 15 shows typical current and voltage waveforms recorded during the experiment on the crossed copper bars. The contact force was chosen at a lower value, a little below *F* = 1 N. Figure 15 also shows a *V*-*I* plot highlighting the change in contact resistance before and after the maximum load current is reached and showing the fritting at the contact location. The voltage at the contacts rises rapidly at first. As the current increases, the temperature of the contact spot becomes extremely high and the voltage drop grows.

A significant break in the voltage waveform appears at *V* = 140 mV. When the current is decreased, the voltage then falls along a different trajectory with a lower slope. Proportionally, the value of the contact resistance is reduced by about 2.65/0.82 = 3.2 times. No welding of the contact surfaces occurred after the weights were removed.

**Figure 16:**
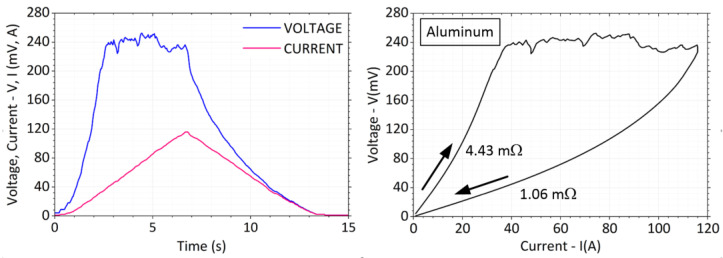
Current and voltage waveform with *V-I* characteristic for aluminum bars.

The fritting voltage for aluminum bars reaches a higher value than for copper, approximately *V* = 245 mV, see Figure 16. Around a current of *I* = 90 A, there is a small drop in voltage and probably a final growth of the contact area.

After the load current is reduced, the voltage falls along a lower curve to a final value of 1.06 mΩ. Proportionally, the value of the contact resistance decreased by about 4.43/1.06 = 4.2 times. No signs of welding were observed after the weights were removed and the contact surfaces were uncoupled.

**Figure 17:**
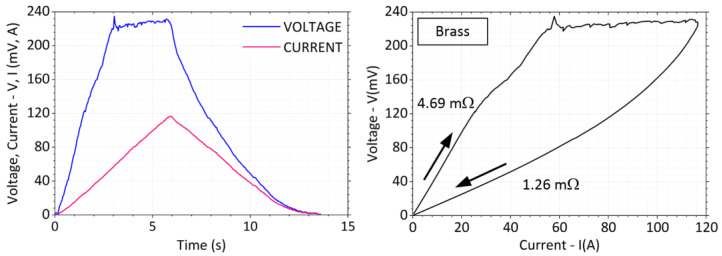
Current and voltage waveform with *V-I* characteristic for brass bars.

During the measurement of brass bars, practically all experiments showed a slight drop in the voltage curve in the range of *V*_1_ = 100 mV, but more often at *V*_2_ = 135 mV. The voltage continued to increase with a slightly lower slope and the final fritting voltage value was then *V*_F_ = 220 mV. Occasionally a small peak occurred almost reaching a magnitude of *V_P_* = 240 mV. A typical waveform of this can be seen in Figure 17.

When the current was further raised, the voltage on the contacts increased only very slowly. After the load current was reduced, the voltage decreased with a lower slope to the final value of the contact resistance of 1.26 mΩ. Proportionally, the value of the contact resistance decreased about 4.69/1.26 = 3.7 times. After the weights were removed and the contact surfaces were uncoupled, there was no sign of welding.

**Figure 18:**
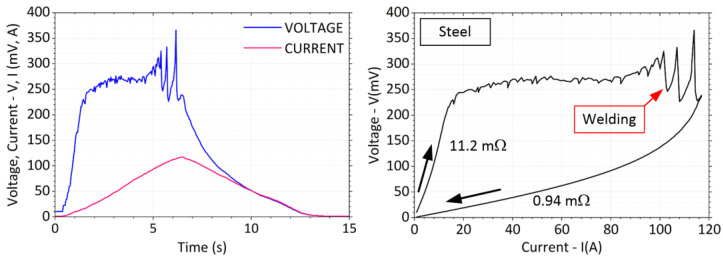
Current and voltage waveform with *V-I* characteristic for steel bars.

In the measurements of the steel cross bars, the voltage on the contacts increased extremely fast already at 10% of the maximum current (*I*_1_ = 12 A), see Figure 18. At a current of approx. *I*_2_ = 80 A there was a violent rise in voltage and subsequent spikes reaching up to *V_P_* = 380 mV in the peak. The voltage spikes continued until the maximum load current was reached without stabilization. The voltage spikes were also characterized by a significant sound effect.

After the load current was switched off and the weights were unloaded, a significant force had to be applied to separate the contact surfaces. From this, it was considered that the welding voltage was achieved in the experiment. The *V*-*I* graph shows a significant change in the slope of the voltage rise. Proportionally, the value of the contact resistance decreased about 11.2/0.97 = 12 times.

## 4. Discussion

Due to the lack of more comprehensive information regarding the design of the contact loading system, we took our approach and designed a simple lever system according to Dr. Holm’s schematics. The loading of the contacts is realized by calibrated weights and the force is measured by a force gauge. As simple as the proposed design is, the overall structural stiffness of the system is not optimal.

The design of the bar holders appears to be problematic, where applied forces *F* > 5 N lead to the bending of the specimen and a reduction in the contact force. For smaller forces *F* < 1 N, on the other hand, the influence of the connection and elasticity of the supply wires causes very poor repeatability of the experiments. This is reflected, among other things, by large deviations of the measured values in the case of smaller applied forces and is evident in all tested samples. The difference between the average and the two extreme minimum and maximum values reaches, for example, ±140% for copper bars and a load force of *F* = 0.5 N and ±200% for a force of *F* = 0.1 N. In the case of aluminum bars, this range of minimum and maximum resistance is smaller, but the contact resistance remains almost constant at both applied forces *F* = 0.1 N and 0.5 N. The above, due to the design limitations, was the main reason for the exclusion of contact resistance values at low values of the load force. The coefficient *K_C_* was then determined from the contact resistance values at load *F* > 1 N.

The use of a frame structure with a crossbeam and vertically acting force, e.g., through a pneumatic cylinder, seems to be generally better than the originally designed system with an aluminum rotating arm and weight. The frame-enclosed construction with a crossbeam (a design well-known from pressing machines) provides better utilization of the applied force at the contact point. Force measurement can be realized by a strain gauge, which can be located under the holder at the bottom of the support. In the case of higher forces, this solution offers compensation for frame deformation, as the strain gauge captures the total resultant force. The expected improvement when using a frame construction is a higher stability of the applied force and a reduction in vibrations and shaking caused by the environment.

Furthermore, research in terms of design limitations has shown that measuring the contact resistance only at selected values of the loading force provides incomplete information regarding the elastic-plastic deformation transition of a-spot. Continuously increasing the loading force and simultaneously measuring the contact resistance in synchronous mode can extremely smooth the results.

The sample holder should be designed so that one side of the sample leans against the base to eliminate its deflection. Otherwise, a part of the contact force is absorbed due to the elasticity of the material. The initially designed mounting, where the sample must be inserted through the holder, is also not optimal. The sample surface may be scratched when being inserted through the holder. Due to its dimensions, it is also difficult to ensure that the current is fed through a conductor with a sufficient cross-section so that it does not affect the sample by its heating. If pads are used along the path of the force transmission to the contact or force gauge to delimit the design tolerances, they must not be elastic (rubber) but rigid, otherwise, the applied force can be absorbed.

Numerical simulation showed that if the supply conductors are attached to the sample at one location, additional deformation of the current lines occurs inside the bar. This deformation, if the sample is short, may further affect the entry of the current lines into the contact point and increase the contact resistance. According to theoretical assumptions, the current lines should be deformed only by the entry into the constriction area of the a-spot without the influence of the current connecting method. It was found that the deformation of the current lines depends on the dimensions of the test sample, most of all on the type of material. The higher the electrical conductivity of the sample material, the faster the imposed deformation decreases by the one-sided input of the load current. FEM simulation models and analysis of the current field can illustrate this phenomenon very well.

In the first part of the work, the basic identification of the material samples was carried out by measuring the specific electrical resistance. Material types were selected which are commonly used for the construction of electrical devices and whose conductivities can be compared with catalog data or data available in the technical literature. Measurement uncertainties were also evaluated for complete identification. The final combined uncertainty with a coverage factor of *k* = 2 was then determined to obtain the resulting specific resistivity of the material. Table 6 shows that the largest uncertainty contribution (53%) is the measurement of the sample diameter *D* followed by the measurement of the current magnitude (37%). The remaining 10% is then accounted for by the uncertainty of the electrode voltage span and DC voltage measurements.

To reduce the uncertainty, the use of a micrometer with a high resolution, e.g., 0.1 μm, seems more appropriate. The ovality of the sample should also be checked with a higher density measurement. Similarly, the higher uncertainty in the current measurement can be attributed to the use of an inexpensive multi-meter, which was only pre-calibrated in the laboratory at a lower current value of *I* = 10 A. On the DC range, the longer measurements showed current drift caused by the internal shunt resistor of the multi-meter. This led generally to the need for long breaks during individual resistivity measurements. For further use, the resistivity measurement has already been optimized and includes the utilization of a calibrated shunt resistor and indirect current measurement using a 24-bit DAQ card.

The main focus of the work was on the measurement of contact resistance as a function of the applied force. All the materials tested showed an enormous influence of chemical layers, which increase the overall contact resistance extremely. During the measurement, the surface layers were not disturbed because the measuring system applies force only in the direction of contact without sliding movement or vibration. This was the reason why the material-dependent *K_C_* factor for Cu was significantly higher than the tabular values used for the design of the power contacts. For power contacts, it is automatically assumed that at least some sliding motion and intensive cleaning of the surface layers occurs when the contact surfaces are coupled. Similarly, surface treatment in the form of brushing could cause an increased contact resistance value.

The influence of the surface layer was extremely pronounced in the case of aluminum bars. The material used is not the direct electrical grade of aluminum but is a structural type with a lower electrical conductivity. During the measurements, it was often the case that an infinite resistance *R_CO_* was measured even with a relatively large applied force *F* > 1 N. No conductive interface formed and the surface layers were able to maintain the set potential difference Δ*V* = 1 V. In many cases, this phenomenon occurred after the contact force had been reduced and then re-applied. A partial improvement in the measurement repeatability was achieved when the aluminum bars were resanded with fine sandpaper at the holder-sample interface and immediately fastened. The tightening of the screws in the holder had to be stronger than for the remaining samples. Overall, it was confirmed that such a holder design is completely inappropriate. In the upgraded design, this solution will be replaced by a holder with several fixing points around the circumference and a tightening belt.

The measurement of the fritting voltage was performed at a lower value of contact force, due to the maximum limits of the current source. With high contact forces or very clean Cu contact surfaces, extremely high current values of *I* > 1000 A are needed to achieve softening voltage. This is also shown in the *R*-*V* diagram of the reference by Dr. Holm and other authors, who adopted it in agreement. For real contacts and safety reasons, the voltage drop across the contacts must be many times lower than the softening voltage of the contact material. The measurements performed at a lower contact force are then an illustration of the classical fritting process of cleaning contact surfaces due to the passage of an electric current.

For alloys such as brass, the grain size of the individual material components is important. The grain size on the surface can be comparable to the size of the a-spot and can affect the contact connection. For example, for an applied force of *F* = 1 N, the a-spot diameter is approximately *2a* = 90 μm according to the parameters of Table 7. The grain size of Cu without heat treatment is in the order of 50 μm. Thus there can be an overlapping of Cu, Zn, and Pb components in the brass alloy and can cause either Zn + Zn or Cu + Cu, or Cu + Zn to be in contact. Then, the breaks in the *V*-*I* curve that are close to the softening voltage of Zn (0.1 V) and Cu (0.12 V) and the welding voltage of Pb (0.19 V) are quite comprehensible.

The steel samples demonstrated significant fritting when low contact forces were applied. The welding voltage was then probably reached just before the maximum load current was reached. Compared to the other tested materials, a significant force (tearing) had to be applied to separate the contact surfaces. This indicates the need for strong contact forces in potential connections with steel conductors and, due to the hardness of its surface, the use of large clamping forces in connections (typically ground rods and grounding systems).

## 5. Conclusions

The purpose of the article was to provide basic information with a more detailed description of the design of a test device for the validation of contact resistance of selected materials used in the design of equipment in electrical engineering.

A set of measurements of specific electrical conductivity (resistivity) of selected materials was performed and as a result, these values were refined. Values of electrical conductivity reported in the technical literature show a higher percentage of variance. The measurements of this material parameter were made concerning the uncertainty determination with an expansion factor of *k* = 2. This type of measurement procedure was created to validate newly developing materials in Al/Cu and rotary swaging combinations that may have a high potential for use in electrical engineering.

The simulation part demonstrated the enormous influence of the deformation of the current lines, neglecting the effect of the position of the input supply electrodes and the method of mounting the measured sample. It was presented that both the material of the holder and the material of the sample and its geometrical dimensions play a role in the distribution of the deformation. Based on the simulation outputs, the overall shape and dimensions of the contact holder were optimized.

The design limitations and flaws of the designed contact resistance measurement system were also found. The knowledge gained from the use of the measurement system will be further applied to the redesign of the new and improved frame structure. The newly developed system will allow additional vertical movement to simulate the sliding effect in the contacts. This could better simulate the real conditions under which the power contacts are switched. This paper shows the importance of measuring the fundamental physical properties of construction materials used in electrical engineering. Typically, the design of devices considers, among other things, the resistance of conductive paths, which in turn determines the power conversion losses and the efficiency of the system. By measuring classical or newly developed materials, we can better search for the appropriate design layout of modern electrical products.

## Figures and Tables

**Table 1:** Values of material-dependent coefficient *K_C_* and shape exponent *n*.

Material	Babikov [8]	Kurbatov [50]	Shape Exponent	
	*K_C_* (μΩ·kg^n^)		Type of Contact	*n* (-)
Copper-Copper	80 ÷ 140	400	Flat—Flat	1
Alumin-Alumin	3000 ÷ 6700	3000 ÷ 6000	Sharp—Flat	0.5
Brass-Brass	670	670	Sphere—Sphere	0.5
Steel-Steel	7600	7600	Brush—Flat	1
LC Copper-Copper	-	90 ÷ 280	Bus Bar	0.5 ÷ 0.7

**Table 2:** Measured and evaluated data for uncertainty Type A for *D* and *L*.

***n***	**Diameter** ***D* (mm)**	**Length** ***L* (mm)**	** 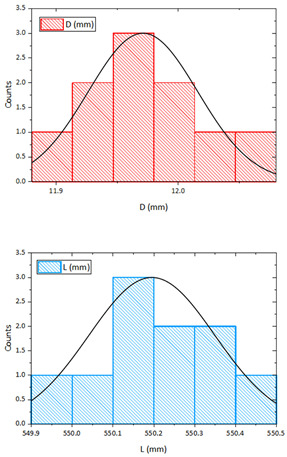 **
1	11.97	550.13	
2	12.03	550.39	
3	11.91	550.41	
4	11.94	550.03	
5	12.05	549.95	
6	12.00	549.93	
7	11.93	550.11	
8	11.95	550.31	
9	12.00	550.27	
10	12.02	550.21	
Arithmetic average	11.980	550.174	
*u_A_* (*D*), *u_A_* (*L*)	0.0148	0.0546	

**Table 3:** Evaluated data for combined uncertainty and coverage factor *k* = 2 for *D* and *L*.

Value	Estimation (mm)	Standard Uncertainty (mm)	Distribution	Coefficient of Sensitivity	Contribution (mm)
*D*	11.980	0.015	normal	1	0.015
Scale *δ*_1_ (*D*)	-	0.012	rectangle	1	0.012
OP infl *δ*_2_ (D)	-	0.012	rectangle	1	0.012
*D*	11.98	-	-	-	0.023
*L*	550.174	0.055	normal	1	0.055
Scale *δ*_1_ (*L*)	-	0.061	rectangle	1	0.061
OP infl *δ*_2_ (*L*)	-	0.060	rectangle	1	0.289
*L*	550.174	-	-	-	0.299
Bar Diameter *D* (mm):		11.98 ± 0.05			*k* = 2
Electrode Spacing *L* (mm):		550.17 ± 0.60			*k* = 2

**Table 4:** Measured and evaluated data for uncertainty Type A for *I* and *V*.

***n***	**Voltage** ***V* (mV)**	**Current** ***I* (A)**	** 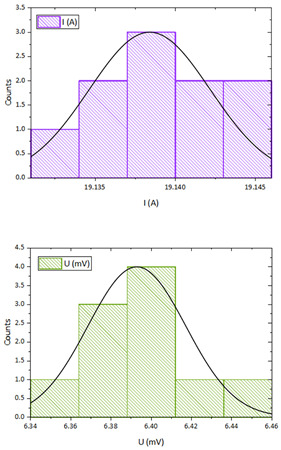 **
1	6.401	19.139	
2	6.396	19.141	
3	6.361	19.135	
4	6.440	19.144	
5	6.414	19.132	
6	6.385	19.140	
7	6.366	19.138	
8	6.389	19.143	
9	6.369	19.137	
10	6.407	19.133	
Arithmetic average	6.393	19.138	
*u_A_* (*V*), *u_A_* (*I*)	0.0077	0.0013	

**Table 5:** Evaluated data for combined uncertainty and coverage factor *k* = 2 for *I* and *V*.

Value	Estimation (A, mV)	Standard Uncertainty (A, mV)	Distribution	Coefficient of Sensitivity	Contribution (A, mV)
*I*	19.138	0.0013	normal	1	0.0013
A-meter *δ*_1_ (*I*)	-	0.0569	rectangle	1	0.0569
*I*	19.14	-	-	-	0.057
*V*	6.393	0.0077	normal	1	0.0077
V-meter *δ*_1_ (m*V*)	-	0.0036	rectangle	1	0.0036
*V*	6.39	-	-	-	0.0085
Current *I* (A):		19.14 ± 0.11			*k* = 2
Voltage *V* (mV):		6.39 ± 0.02			*k* = 2

**Table 6:** Evaluated data for uncertainty of specific resistivity of sample with coverage factor *k* = 2.

Value	Estimation	Standard Uncertainty	Sensitivity Coefficient	Uncertainty Contribution
*D*	11.98·10^−3^ m	22.2·10^−6^ m	11·10^−6^ Ω	244·10^−12^
*L*	550.17·10^−3^ m	300·10^−6^ m	–124·10^−9^ Ω	–37·10^−12^
*I*	19.14 A	0.057 A	–3.6·10^−9^ Ω·m/A	–205·10^−12^
*V*	6.39·10^−3^ V	8.5·10^−6^ V	11·10^−6^ m/A	94·10^−12^
*ρ*	68.41·10^−9^	-	-	334·10^−12^
Resistivity *ρ* (nΩ·m):		68.41 ± 0.67		*k* = 2

**Table 7:** Basic material parameters of samples for contact resistance measurements.

Material	Name	Resistivity *ρ* (nΩ·m)	Reference *ρ_D_* (nΩ·m)	Modulus of Elasticity *E* [GPa]
Copper	Cu-ETC	17.57 ± 0.24	16.5 ÷ 18	117 [56]
Aluminum	AlCu4PbMg	47.91 ± 0.51	45 ÷ 49	74 [57]
Brass	CuZn39Pb3	68.41 ± 0.67	67 ÷ 58	96 [58]
Steel	S235JR	149.70 ± 1.40	140 ÷ 150	210 [59]

**Table 8:** Values of material-dependent coefficient *K_C_* and shape-exponent *n*.

Material Combination	HOLM (*n* = 1/3)	Experimental Values	
	*K_TE_*	*K_EX_*	*n*
	(mΩ·N^n^)	(mΩ·N^n^)	(-)
Copper-Copper	0.213	0.51	0.43
Alumin-Alumin	0.499	4.93	0.40
Brass-Brass	0.776	1.95	0.39
Steel-Steel	2.344	13.4	0.47

## Data Availability

Not applicable.
